# American Cyclopædia of Practical Medicine

**Published:** 1836

**Authors:** 


### Art. IX.—The American Cyclopædia of Practical Medicine and Surgery.

*The American Cyclopædia of Practical Medicine and Surgery, a Digest of Medical Literature.* Edited by Isaac Hays, M. D., Part x. Philadelphia, September, 1836.

This number closes the second volume of the above work. The articles of which it is composed, are interesting and well written, and the work certainly deserves the support of the profession. It is, to a great extent, American and hence, I hope, it will be well sustained. The editor informs his subscribers, however, that such is not the fact. From his statement it appears that the subscription has, thus far, fallen short of the actual expenses. Of course the work must stop unless the patronage is increased. Those who do not take it already should do so immediately, and those who do should use their efforts to get others to do likewise. The volumes already published, contain a number of rare and useful articles complete within themselves, and hence they will form a useful addition to our medical libraries, should the work proceed no farther. It has always been favorably noticed in this Journal, and the editors are pleased to find that it has received the same commendation, from critical authorities, both at home and abroad. It is, however, a lamentable fact that the American periodicals in medicine do not receive the support to which they are entitled. My experience proves clearly to my mind that but few of them pay the expenses of publication, and hence they should only be undertaken by those who choose to labor for the elevation of the profession or for the support of medical institutions to which they are attached.

That the readers of the Journal may see that the Cyclopædia is fully sustained in its course, I will present them with an extract on the atmosphere from the prolific pen of professor Dunglison:

"*Atmospheric Moisture.*—There is another condition of the atmosphere, which—singly or combined with the others we have mentioned, exerts considerable influence over the functions. We allude to the *Hygrometric.*

As air possesses the property of dissolving water, all liquid bodies, when exposed to it, experience a certain degree of evaporation; the amount of such evaporation varying according to the degree in which water is already contained in the air.

It has been a question with physiologists, whether the air abstracts moisture from the animal body as it does from inorganic substances. They who think that the cutaneous and pulmonary transpirations are mere transudations, or dependent upon a physical permeation of fluid from within the appropriate vessels to without, and independently of all vital agency, believe in the affirmative; whilst they who regard those transpirations as altogether vital, consider that no such physical effect can result. Others, again, with more propriety, believe that the condition of the external air may concur in modifying the exhalation, even if it be regarded purely vital.

The supporters of the first opinion adduce the instances of fishes, which, if taken out of their proper medium, and kept for some time in the air, lose a considerable portion of their weight by this kind of evaporation, or transudation. M. Edwards affirms, that having endeavored to prevent evaporation by placing a cold-blooded animal in a moist atmosphere, and at a temperature equal to that of the animal, so as to reduce the transpiration to that which was accomplished organically, or by the vital action of secretion, he found that the physical evaporation formed five-sixths of the ordinary loss by transpiration. Adelon, however, objects to any inference deduced from aquatic animals being applied to man;—"the former," he remarks, "are impregnated with water, and as soon as they are exposed to the air, permit it to transude." But such, he affirms, is not the case with man, in whom it is necessary, that the matter of transpiration should be secreted by appropriate organs, and be deposited on the pulmonary and cutaneous surfaces. He denies, also, that anything like physical permeation takes place in the living body. In another work, however, (*Human Physiology*, 2d edit. I. 42.) we have endeavored to show, that the living tissues are penetrable, and that both imbibition and transudation takes place in the living body. This, we think, is indisputably proved. But even were we to grant the position assumed by Adelon,—that the cutaneous and pulmonary traspirations are produced by vital agency alone, and in no respect to be assimilated to physical transudation, a great agency in modifying the quantity of these transpirations must be ascribed to the varying condition of the atmosphere as regards moisture. If the air be dry, its power of absorption is greater; the perspirable matter evaporates as soon as it is secreted; but when the air contains much moisture, the perspirable matter does not readily evaporate, but accumulates on the surface in a sensible state. In the former case, we should expect the activity of the exhalants to be increased by the ready removal of the secretion, and in the latter to be diminished, for opposite reasons.

It is not probable however, that the main effect is induced in this way, but that the process is of a more physical nature, and that the body parts with the watery fluids, contained in the vessels, by simple transudation, although we are not prepared to deny, that a part of the result may be produced on the secretory vessels in the mode mentioned.

From what has been remarked, it can be easily understood why, in a warm moist air, we seem to perspire more than in a hotter and dryer, although we may really be exhaling less. It is asserted by Schmidtmyer, that in the climate of Chili, notwithstanding the very high temperature in summer, the perspiration passes off so entirely in the insensible form, that, during the most violent exercise, it might be doubted whether any perspiration whatever exists.

If the air be greatly charged with moisture, especially during the heat of summer,—owing to a diminution of the cutaneous and pulmonary transpiration, the evaporation of which constitutes a cooling process, we feel languid and listless, with an indisposition to all mental and corporeal exertion. This is the cause why we suffer little more during the hot summers of this country, than in those of Great Britian, where the air is always more loaded with humidity, although the thermometer may be 15° or 20° higher here than there.

Again, when we are exposed to a moist temperature much greater than that of the body, we may seem to perspire profusely, whilst the cutaneous moisture may be chiefly owing to another cause.

In certain experiments instituted by Dr. Geo. Fordyce and Sir Charles Blogden, with heated air, they found in a temperature of 260° of Fahrenheit, that small quantities of water in metallic vessels speedily boiled, and that streams of moisture poured down the whole surface of the body; but that this was merely the vapor of the room, condensed by the cooler skin, the temperature of which was only raised a few degrees above the ordinary standard, was proved by the fact, that when a Florence flask, filled with water of the same temperature as the body, was placed in the room, the vapor condensed in like manner upon its surface, and ran down in streams.

On the other hand, when the air is cold and moist,—owing to aqueous vapor being a better conductor of caloric than air, the heat is abstracted in greater quantity from the frame, and we feel more chilly than the temperature, it would seem, is calculated to explain; and therefore more liable to have those disordered and irregular actions of the capillary system induced, which give occasion to different febrile and inflammatory disorders.

M. Edwards ascribes the uneasy sensations, experienced on the tops of lofty mountains, to the augmented evaporation from the lungs, produced by diminished atmospheric pressure, the great dryness. This great dryness of the air at lofty elevations was appreciated by Garcilasso de la Vega. "It is a well-known fact," he observes, "that the Adelantado Don Diego de Almagro, on his march towards Chili, when, as is probable, he was led by his guides over the highest plain of Tacora, lost more than 10,000 Indians, 150 Spaniards, and a number of horses, all of whom fell a sacrifice to hunger, thirst, and this disease. The soldiers in that memorable expedition built themselves walls of the dead bodies of their comrades, merely to protect themselves against the drying effect of the wind."

But facts as M. Edwards judiciously observes, connected with an excessive evaporation from the lungs, may be observed in other than elevated regions.

In winter, when, during a very sharp cold, an apartment is warmed by means of a stove, a painful sensation is experienced by many persons in the chest. The air, in a frost, contains scarcely any watery vapor, and the heat of the stove, by augmenting the temperature of the air, increases its capacity for vapor, so that a much greater evaporation is produced than in summer. It is an old custom to place upon the stove a vessel of water to remedy the inconvenience, and the practice is attended with advantage.

It is probably by this abstraction of the moisture from humid bodies, that air acts as an irritant to wounded and ulcerated surfaces (see *Air*,) and the great improvement which has taken place in the management of such cases has consisted in carefully excluding air, the admission of which occasions a rapid evaporation of the moisture covering them, and excites irritation in the vessels, whose office it is to effect the reparatory process.

The barometric and thermometric influence of the air is exerted with more or less energy upon the animal frame, according as its hygrometric condition is more or less considerable; that is, according as it is more or less dry or damp.

We have seen that the sensations of heat and cold, which we experience from the air, are greater when the air is damp, owing to the presence of water between its particels adding to its conducting power; and lastly, that as the dissolving power of the air augments in proportion to its dryness and temperature, its action upon the fluids of the body must be less in a moist than in a dry atmosphere.

It may be remarked, by the way, that a moist atmosphere is better adapted than a dry one for dissolving various animal, vegetable, or mineral substances, which are susceptible of volatilization. We have many instances to prove, that volatilizable substances are sooner converted into the gaseous state under such circumstances. Camphor is found to votalize with much greater celerity in damp situations, and every one has noticed the fragrance of a garden after a summer's shower. There are certain bodies, too, which require the presence of moisture for their escape. Thus, the odorous particles of argillaceous substances are quiescent, until they are breathed upon, or, in other words, become moistened by the fluid from the lungs, or by moisture of some kind, after which the mineralogist readily recognizes their characteristic odor.

Every one must have noticed how powerfully the stench of putrid ditches is conveyed to the olfactory organs in summer, previous to rain, when the air becomes charged with moisture; and how readily offensive substances are detected in a fog by the same sense.

The agency of moisture is, doubtless, also concerned in the conveyance of the various emanations from the soil, that produce endemic disease. It has long been noticed, that whilst the inhabitants of a plain, on the level of a marshy land, have escaped those diseases that are known to be produced by the emanations from such land,—or by *malaria* (q. v.,) as it has been termed by the Italians,—those dwelling on neighboring elevations have suffered extensively. Observation would seem to have shown, that this malaria is somewhat heavier than atmospheric air; but as watery vapor is incessantly exhaled from the surface of the earth under the influence of solar heat, and as this vapor possesses so little specific gravity, it takes up the miasmata along with it, and under favorable circumstances, they impinge on these elevations.

The same reasons apply to the communication of the matter of contagion, which would appear to be modified in its activity by the degree of moisture in the atmosphere influencing its solubility and volatility; but on this topic our evidence is not quite as satisfactory. The same may be said of epidemic influences, of which our ignorance is unhappily so profound. It may be remarked, however, as some corroboration of this view, that the Harmattan, a wind which blows periodically from the interior of Africa towards the Atlantic Ocean, and which is characterized by its extreme dryness, is asserted put an end to all epidemic and contagious affections—even to smallpox; and it is said that, at such times; infection is not easily communicable by art. We shall find, hereafter, that humidity modifies the action of atmospheric electricity on the animal body, as well as the electrical condition itself.

*Atmospheric Vicissitudes.*—It has been already seen, that in the varying atmospheric conditions, which have been considered, the system has the power of accommodating itself to the changes, provided they are not too extensive or sudden. But if the mercury were to vary at once from 28 to 31 inches, or conversely, it is difficult to say what might be the extent of the effects of such sudden vicissitude. Or, again, if the temperature should suddenly rise from—55° of Fahrenheit, to + 130, as a natural consequence of this rise the barometer would fall, and from these combined causes—even from the vicissitude of temperature taken singly—man might cease to exist.

Vicissitudes in the hygrometical state of the atmosphere would probably be borne with the greatest impunity.

It can rarely happen, that these vicissitudes in the barometric, thermometric, or hygrometric conditions of the air, are experienced singly. It has already been seen that as we ascend in the air, the atmosphere necessarily becomes lighter, the mercury of the barometer consequently descends, and, at the same time, greater and greater coldness is experienced according to the elevation, so that if we are ascending high mountains we ultimately attain the regions of perpetual congelation. We have seen, also, that at very great elevations, the air is much dryer, and that inconvenience is actually sustained from this cause. High up in the atmosphere, we have, consequently, a combination of a low state of barometric, thermometric, and hygrometric conditions.

Warm air, again, being more expanded, the barometer sinks in it, whilst a larger quantity of aqueous vapor can be held in the invisible state than when the temperature is lower.

These facts show, that the different atmospheric modifications, which we have considered, may be variously circumstanced so as to give rise to much of that peculiarity which we notice in different climates, and to the various mutations experienced in the air of the same district of country.

Vicissitudes in temperature are the most appreciable by our senses, and to them, consequently our attention is most frequently directed. A rapid alternation from heat to cold is felt the most disagreeably, and we are disposed to refer numerous morbid conditions to it, especially if the cold be attended with dampness, which it is sure to be, if the vicissitude has been very sudden.

During the state of warmth, a large quantity of vapor may be retained in the air in an insensible form, which becomes apparent if the temperature suddenly subsides to an unusually depressed point.

Robust individuals may experience such alternations without detriment; but the delicate,—they who are liable to internal affections on slight irregularities often suffer greatly. It has been supposed that much of this effect is owing to a sudden check to perspiration, and this may account for the mischief in many cases, but unless the change be remarkably rapid, the system will generally accommodate itself, so that the depuration, previously accomplished by the skin, may take place to a considerable extent from the urinary organs; for we have already seen, that in the winter months this depuration exceeds the cutaneous, whilst the contrary obtains in summer. Thus, the air may continue, as it often does in winter, in this climate, for days together, largely below the freezing-point, and yet no evil, under the ordinary precautions, may result from protracted exposure to it.

It does not, indeed, appear probable; that many of the maladies so often ascribed to depressed temperature or to taking cold, are owing to mere diminution in the *general* cutaneous exhalation, but rather, that they are ascribable to local irregularities of the capillary system, between all the parts of which there is such an extensive and intimate sympathy, that if one part be irregularly affected, another portion of the system, more disposed than the rest, owing to inappreciable circumstances, to assume morbid derangement, becomes implicated.

The probability of some evil, resulting from getting the feet wet, is proverbial; yet the effect, immediately produced by exposure, involves but a slight extent of surface. Still, if twenty people be exposed to this cause of disease, upwards of two-thirds will probably be attacked with inflammation or irritation somewhere. One may have one form of catarrh; another may have a second; another inflammatory sore throat; another pneumonia; another inflammation of the bowels, and so on; according as the capillary system of one part, in the particular individual, is more liable, at the time, to take on an increased action than the rest.

It has been generally asserted by writers, that a sudden vicissitude from heat to cold is likely to affect the bowels by *driving in* the perspiration, and occasioning it to settle on the mucus membrane of the intestinal tube. This does not appear to us to be good philosophy, although the fact of diarrhœa supervening under such circumstances may be indisputable.

We have seen, that the mucus membranes essentially resemble the skin in function, but they differ greatly in one respect. The cuticular covering of the skin impedes the absorption of substances from without, whilst the mucus membrane of the intestinal canal has the important office of absorbing all substances possessed of the necessary degree of tenuity. It is more an absorbing than an exhaling membrane. It is probable, therefore, that where a bowel-affection results from exposure to cold, under the circumstances mentioned, the excited action of the exhalants is caused by the lining membrane of the intestines sympathizing with the irregular action of the cutaneous capillaries, so that the membrane is not in a simple state of exhalation, occasioned by the *driving in* of the cutaneous transpiration, but actually labors under inflammatory excitement or irritation; for such is always present to a greater or less extent in these cases—produced in the same manner as where a distant organ becomes irritated in consequence of an irregular action of the capillaries of the feet, in the case assumed above.

It need scarcely be remarked, that these effects, as well as all those that are produced by atmospheric vicissitudes, affect the feeble, and the convalescent, and those debilitated and irritated by previous evacuations or disorders more than the healthy."

